# A Sensitive and Rapid Method for Detecting Formaldehyde in Brain Tissues

**DOI:** 10.1155/2017/9043134

**Published:** 2017-09-24

**Authors:** Xiangpei Yue, Yaoyue Zhang, Wen Xing, Yutong Chen, Chenyang Mu, Zhan Miao, Peichun Ge, Tingting Li, Rongqiao He, Zhiqian Tong

**Affiliations:** ^1^Alzheimer's Disease Center, Beijing Institute for Brain Disorders, Capital Medical University, Beijing 100069, China; ^2^Beijing No. 12 Laboratory of Brain and Cognitive Sciences, Beijing 100071, China; ^3^State Key Laboratory of Brain and Cognitive Sciences, Institute of Biophysics, Beijing 100101, China

## Abstract

The existing methods for detecting formaldehyde (FA) in brain samples are expensive and require sophisticated experimental procedures. Here, we established a highly sensitive and selective spectrophotometric method, which is based on a reaction in which FA reacts with colorless reagent 4-amino-3-penten-2-one (Fluoral-P) to produce a yellow compound, 3,5-diacetyl-1,4-dihydrolutidine (DDL), which can be detected by a spectrophotometer at 420 nm at room temperature. The sensitive response time point was found to be at the first hour, and the optimal pH of derivative reaction was pH 6.0. The limit of detection (LOD) and the limits of quantization (LOQ) for detecting FA were 0.5 *μ*M and 2.5 *μ*M, respectively. Using this method, an abnormally high level of FA was detected in both the brains of FA-injected mice and autopsy hippocampus tissues from patients with Alzheimer's disease. This finding suggests that the modified Fluoral-P method is effective for measuring levels of FA in the brains.

## 1. Introduction

Formaldehyde (FA) is an industrial chemical product that is widely used to manufacture building materials and household products and is released from several sources within indoor environments, such as particle board, household products, and plywood [1]; hence, a large number of workers and inhabitants are inevitably exposed to FA [2]. Initial studies showed that gaseous FA could cause carcinogenic and fetal abnormalities [3], and clinical and epidemiological investigations have found that work-related exposure to FA results in headaches, anxiety, fatigue, sleep disorders, in particular, cognitive disorders [4, 5]. In addition, the results of animal experiments have consistently revealed that gaseous FA exposure induced abnormal behaviors, including spatial memory deficits [6–8].

Interestingly, abnormal levels of endogenous FA have been detected in humans exposed to exogenous FA [9], as well as in aged mice and rats [10], in healthy older adults with cognitive disorders [11], and in patients suffering from Alzheimer's disease (AD) [12]. Documentary evidence indicates that a direct intracerebroventricular injection of excess FA into animal models causes central nervous system damage and, in particular, a marked impairment in memory [13–16]. These data strongly support the notion that excess FA in the hippocampus leads to cognitive deficits.

In view of the neurotoxic effects of FA, various analytical methods have been developed to detect FA in animal tissues. The earliest determination method for measuring FA in brain tissues (~0.16–0.24 mM) was carried out using gas chromatography/mass spectrometry (GC–MS) [17]. Another sensitive method, developed for the detection of FA in the liver, utilized high-performance liquid chromatography with an ultra violet detector (HPLC-UV) [18, 19]. More recently, using a high-performance liquid chromatography with a fluorescence detector (HPLC-Fluo), we found that there was 0.25–0.32 mM FA in the hippocampi of normal adult mice and rats [20]. Although these existing approaches provide some accurate and ultrasensitive assays for FA detection in the brains, several disadvantages, such as expense, sophisticated experimental procedures, and noxious analytical reagents, have limited their practical applications. Therefore, a simple, sensitive, and efficient method for determining trace amounts of FA in biological samples is needed.

Herein, we report that a highly sensitive and selective spectrophotometric method for detecting FA in the brains of mice at room temperature has been established using a colorless reagent- 4-amino-3-penten-2-one (Fluoral-P) which selectively reacts with FA to produce a colored compound, 3,5-diacetyl-1,4-dihydrolutidine (DDL). Thus, this modified Fluoral-P method is suitable to detect brain FA and assess FA neurotoxicity.

## 2. Materials and Methods

### 2.1. Reagents and Chemical FA Solution

37% (*w*/*v*; Sigma-Aldrich, Saint Louis, USA); 4-amino-3-penten-2-one (Fluoral-P, CAS: 1118-66-7. http://TCIchemicals.com, Japan); hydrochloric acid (HCl, 1 N, Sigma-Aldrich, Saint Louis, USA); sodium hydroxide (NaOH, 2 N, Sigma-Aldrich); dimethyl sulfoxide (DMSO, Sigma-Aldrich, Saint Louis, USA); trichloroacetic acid (TCA, Sigma-Aldrich, Saint Louis, USA); deionized water (Milli-Q water); phosphate-buffered saline (PBS, 1 mM, Beijing Institute of Chemical Technology, China); and normal saline (NS, Beijing Institute of Chemical Technology, China) were used in the study.

### 2.2. Apparatuses

Refrigerator (Haier Group, Beijing, China); pH meter (PHS-3C, Shanghai instrument electric science instrument Limited by Share Ltd., Shanghai, China**)**; adjustable high-speed homogenizer (FSH-2A, Liangyou Experiment Instrument Factory, Changzhou, China); Model ZD2011584 from Millipore (Nepean, Ontario, Canada); SP-Max 2300A multiskan spectrum microplate spectrophotometer (Flash spectrum of Shanghai Biological Technology Co. Ltd., Shanghai, China); and Morris water maze (Shanghai Biowill Co. Ltd., Shanghai, China) were used in the study.

### 2.3. Preparation of Chemical Solutions

FA stock solution (100 mM, 12.3 mL): FA solution (0.1 mL, 37%) was pipetted into 12.2 mL of PBS (1 mM, pH at 4.0, 5.0, 6.0, and 7.0, resp.). FA working solutions were 0.001, 0.01, 0.1, 1, and 10 mM, pH at 4.0, 5.0, 6.0, and 7.0, respectively. Fluoral-P store solution (500 mM): 0.05 g was dissolved in 1 mL DMSO and stored in an ice-box at 4°C for not more than 2 months. Fluoral-P working solutions were 0.5, 5, and 50 mM, pH at 4.0, 5.0, 6.0, and 7.0, respectively. Trichloroacetic acid (10%, *w*/*v*) was made in PBS (1 mM, pH 6.0) and kept in the dark.

### 2.4. Brain Tissue Homogenates and Extraction

Following the Morris water maze test, all mice were anesthetized with pentobarbital sodium (10 mg/kg, intraperitoneally (i.p.)) and then sacrificed by cervical dislocation. Brain tissues were immediately removed with medical scissors, rinsed in ice-cold PBS, and then homogenized in 0.1 mM PBS (pH 6.0) at a ratio of 1 : 4 between brain weight and PBS volume. The brain homogenates were added into 10% TCA solution at a volume ratio of 1 : 2. Finally, the mixtures were centrifuged at 12,000 ×g for 10 min at 4°C and supernatants were frozen at −80°C until further use.

### 2.5. Protocol of Brain FA Analysis

#### 2.5.1. Optimal pH and Time of Derivative Reaction

To determine the optimal pH value of derivative reaction between Fluoral-P and FA at room temperature, five 0.1 mL aliquots of different concentrations of FA standard solutions (0.001, 0.01, 0.1, 1, and 10 mM, pH at 4.0, 5.0, 6.0, and 7.0, resp.) were added to five vials in order to prepare a series of calibration standards. In addition, 0.8 mL of PBS (pH at 4.0, 5.0, 6.0, and 7.0, resp.) was pipetted into a separate vial after which aliquots of 0.1 mL 5 mM Fluoral-P solution (pH at 4.0, 5.0, 6.0, and 7.0, resp.) were added to each of the five vials. These mixtures were pipetted into 96-well plates and incubated at room temperature for 0, 15, 30, 60, 120, and 180 min, respectively, and the intensity of the colored derivative, DDL, was quantified by a spectrophotometer at 420 nm.

The calibration curves which covered a FA concentration range of 0.001–1 mM were prepared. For routine analysis, a one-point calibration in duplicate was prepared daily and used for quantitative calculation of brain samples.

#### 2.5.2. Variations of Within-Day and Day-to-Day

Control brain samples (*n* = 9) were analyzed for DDL intensity on day 1 to evaluate the variation in within-day FA concentrations at 0, 15, 30, 60, 90, 120, and 180 min, respectively. Another group was assayed to evaluate the variation in day-to-day FA concentrations, on day 1, 7, and 14, respectively.

### 2.6. Animals

All specified pathogen-free adult male C57BL/6 mice (*n* = 36, 6 weeks old, 18–20 g) were provided by Capital Medical University Laboratory Animal Resources (Beijing, China) and housed in standard conditions (12 h light–dark cycle, 70%–80% humidity, 25 ± 1°C), and food and water were provided ad libitum. All animal experiments were conducted in accordance with the National Institutes of Health Guide for the Care and Use of Laboratory Animals and were approved by the Office of Scientific Research Management of Capital Medical University, Beijing, China (AEEI-2015-032).

### 2.7. FA Metabolism after FA Injection and Morris Water Maze Test

Eighteen mice were used to observe FA metabolism in the brains of normal adult mice that were administered an intraperitoneal injection of either FA (i.p., 0.6 mM, 0.5 mL) or normal saline (0.5 mL) at 30 min. In addition, 18 mice were used for Morris water maze test. Nine mice were injected with FA (0.6 mM, 0.5 mL; i.p.) for 7 consecutive days, 30 min prior to each consecutive maze evaluation. Control mice (*n* = 9) were i.p. injected with normal saline (0.5 mL) and underwent the same experimental procedures as above. All spatial trainings and memory retrieval experiments involving the Morris water maze were conducted as previously described [12, 13].

### 2.8. Autopsy Sample of Human

Autopsy hippocampus tissues from healthy age-matched controls with ApoE^ε3/ε3^, and AD patients with ApoE^ε4/ε4^ genotypes were provided by the Netherlands Brain Bank (NBB) (Supplementary Table 1 available online at https://doi.org/10.1155/2017/9043134). There were no significant differences between the groups with respect to demographic data.

### 2.9. Statistical Analysis

Graphs were generated using GraphPad Prism version 5.01 (GraphPad Software Inc., San Diego, CA, USA). Statistical analyses were generated using SPSS software version 21.0 (SPSS Inc., Chicago, IL, USA). In the Morris water maze experiment, Fisher's least significant difference was used for post hoc comparisons, and the difference between the different treatment groups within each consecutive day was analyzed by using a one-way ANOVA. For other experiments, statistical significance was determined by using a one-way ANOVA followed by Tukey's post hoc test. The data is represented as mean ± standard error over three independent experiments. *P* < 0.05 was considered statistically significant.

## 3. Results

### 3.1. The UV Absorption Peak of FA, Fluoral-P, and DDL

**Figure 1:**
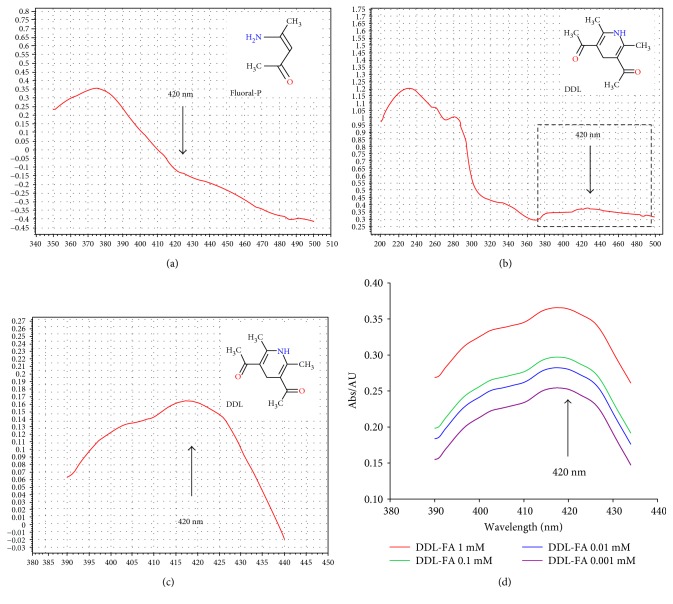
The spectrograph of Fluoral-P (a), DDL (b), and the amplified spectrum of DDL (c). The dose-dependent UV absorption peak of DDL at 420 nm derived from the reaction between Fluoral-P (0.5 mM) and FA (d) (0.001, 0.01, 0.1, and 1 mM, resp.).

To confirm the proposed mechanism of the derivative reaction between two colorless reagents (FA and Fluoral-P produce a colored compound, DDL [21, 22]), we first observed the changes in the color of the mixture between Fluoral-P and FA. Soon after the addition of Floural-P, a yellow DDL was generated (Supplementary Figure 2), and the UV absorption peaks were determined. Results showed that there was not an obvious absorption peak at 420 nm in the colorless Fluoral-P solution (Figure 1(a)), but following an addition of FA solution, a marked peak at 420 nm was sensitively detected at several seconds (Figures 1(b) and 1(c)). Notably, there was a dose-dependent absorbed peak of DDL (420 nm) formation between Fluoral-P (0.5 mM) and FA at different concentrations (Figure 1(d)). These data suggest that the FA derivative, DDL, is a candidate for spectrophotometric detection of FA.

### 3.2. Optimization of the Reaction Condition for FA Derivative—DDL

**Figure 2:**
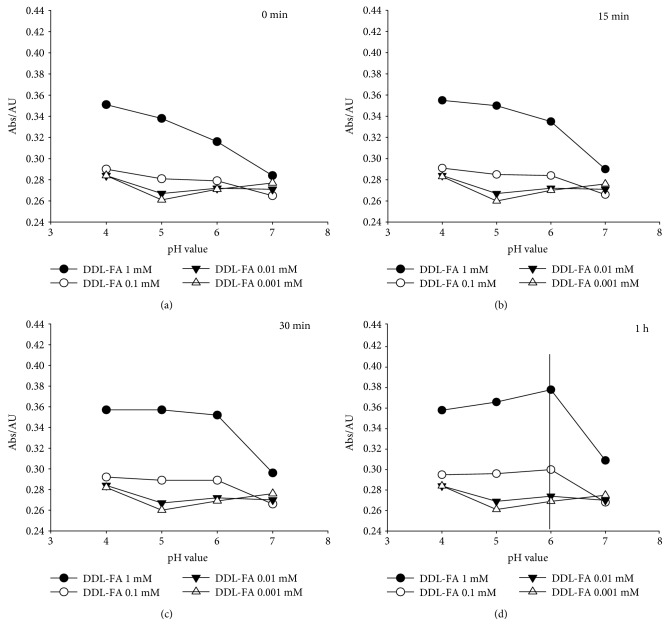
Changes in the UV absorption peak of DDL at 420 nm following the reaction between Fluoral-P (0.5 mM) and FA (0.001, 0.01, 0.1, and 1 mM), after incubation at 0 min (a), 15 min (b), 30 min (c), and 60 min (d), respectively.

To obtain high sensitivity for this method, we explored a number of experimental parameters, such as reaction pH, reaction time, and reaction temperature. First, the effects of pH on the formation of DDL at different times were investigated during the derivative reaction. The results showed that UV absorption values of DDL at 0, 15, and 30 min were gradually decreased with an increasing range of pH (pH 4.0–7.0) of the Fluoral-P/FA mixture at room temperature (25 ± 1°C) (Figures 2(a), 2(b), and 2(c)). Interestingly, the absorption values of DDL were increased to a maximum of 0.379 at pH 6.0, but markedly decreased after the first hour (Figure 2(d)).

**Figure 3:**
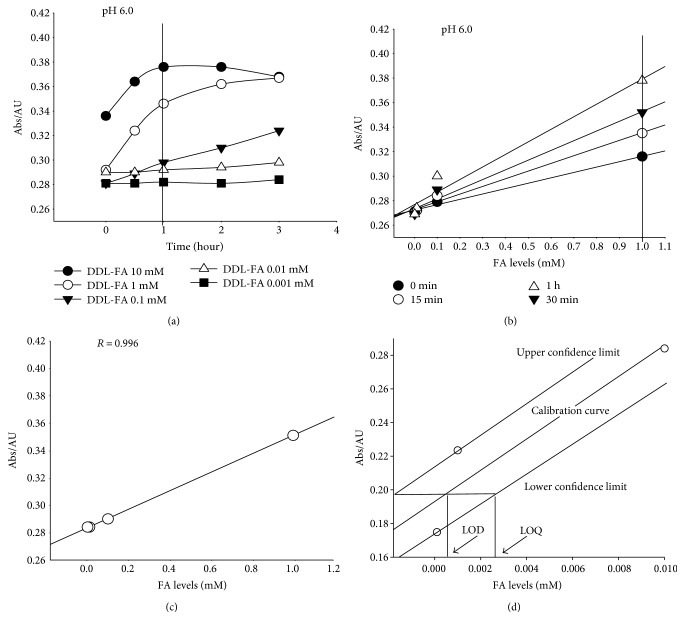
Determination of the response times at the optimized pH 6.0 (a), the different standard curves of DDL at different times (b), the standard curve at 1 h (c), and the LOD and LOQ of this spectrophotometric method at 1 h (d).

The standard curves of DDL derived from FA (0.001, 0.01, 0.1, and 1 mM, pH 6.0) and Fluoral-P (0.5 mM, pH 6.0) at 0, 15, 30, and 60 minutes, respectively, were determined to be linear (*R* > 0.999), but there was a significant difference among the slopes of four regression equations (Figures 3(a) and 3(b)). These results suggest that the optimal conditions for the derivative reaction of DDL are pH: 6.0; reaction time: 1 h; and reaction temperature: room temperature.

### 3.3. The Limit of FA Detection

Next, we determined the upper limit of FA detection from this method with results indicating that an absorbance of 420 nm was found when Fluoral-P (0.5 mM, pH 6.0) was added to solutions (pH 6.0) at or equal to 10 mM FA for 1 h. Any concentrations higher than 10 mM (at optimal conditions) FA did not produce a linear change in UV absorbance of 420 nm over the first hour (Figures 3(a) and 3(b)), thus suggesting that the upper limit of FA detection for using this method is 10 mM.

The actual concentrations of FA at pH 6.0 within the standard curve at 1 h were 0.001, 0.01, 0.1, and 1 mM, respectively. The regression equation of the calibration curve was *y* = 0.067 and *x* + 0.283 with a linear regression coefficient (*R*) of 0.999 (Figure 3(c)). In the routine analysis, a one-point calibration in duplicate could be used instead of a calibration curve, but a new calibration curve was prepared to check the instrumental system in case of any deviation. The limit of detection (LOD) and the limit of quantization (LOQ) were calculated from the confidence intervals of the regression line of the calibration line following the method as previously described [23–25]. The LOD and LOQ of the method were 0.5 and 2.5 *μ*M, respectively (Figure 3(d)).

### 3.4. The Repeatability and Stability of the Method

Nine replicates of control brain samples were analyzed at various time points in a day to evaluate within-day variations. The same samples of above brain homogenates were also analyzed on different days to evaluate day-to-day variations. The data of variations of within-day are summarized in Table 1. These data indicate that there was no difference in concentrations of FA in brain samples within a day (average levels: 0.2705 ± 0.0045 mM; *P* > 0.05). The results of variation of day-to-day showed that the average relative standard deviations were less than 5% (Table 2). These data indicate that this method can accurately and precisely determine concentrations of FA in the brain samples.

### 3.5. Analysis of Brain FA and Assessment of FA Neurotoxicity

**Figure 4:**
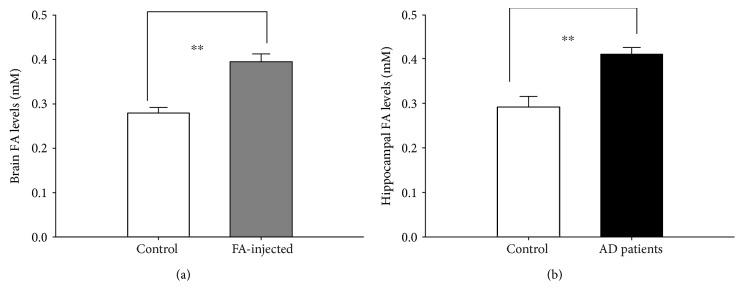
Changes in the brain FA concentrations of the FA-injected mice (a) and autopsy hippocampal tissues from AD patients (b).

Finally, to investigate whether this method can assess the changes in FA levels in the different brains samples, we detected FA contents in the brains of FA-injected mice after intraperitoneal injection of FA 30 min (*n* = 9) and these autopsy hippocampal tissues from AD patients (*n* = 9) and age-matched controls (*n* = 8). The results showed that there was a marked elevation in brain FA levels in the FA-injected mice compared with the control mice (con groups: 0.279 ± 0.012 mM; FA groups: 0.395 ± 0.017 mM; *p* < 0.01) (Figure 4(a)). More importantly, these FA-injected mice associated with higher levels of FA exhibited more severe impairments in memory behaviors in Morris water mazes than the control mice (Supplementary Figure 1). Moreover, FA concentrations in autopsy samples from AD patients were significantly higher than those of age-matched controls (con groups: 0.286 ± 0.021 mM; AD groups: 0.420 ± 0.013 mM; *p* < 0.01) (Figure 4(b)). These results indicate that our novel new spectrophotometric method is suitable to detect FA concentrations in the brain and suggesting that excess FA indeed induces cognitive impairments.

## 4. Discussion

Several decades ago, the colorless reagent, Fluoral-P, was confirmed to be a derivative reagent for the rapid detect FA in waste water, indoor air, and liquor [22, 26, 27], but to our knowledge, was never used to measure FA in brain tissues. In the present study, a highly selective spectrophotometric method using Fluoral-P, which selectively reacts with FA to produce a colored compound-DDL for the sensitive determination of FA in brain samples, was developed. Compared with GC/MS and HPLC methods, the sample preparation process and derivative of the reaction method were relatively simple and straightforward. Herein, this spectrophotometric method is a promising and potential practical application for detecting FA in the brains.

Substantial evidence has shown that gaseous FA can induce cognitive impairments in animals and humans [5, 8]. During aging, chronic accumulation of endogenous FA is thought to be a risk factor for sporadic age-related dementia [10, 28]. Furthermore, excess FA has been found to contribute to the pathological aggregation of amyloid fragments and tau hyperphosphorylation in normal adult mice and monkeys [29, 30]. More importantly, a direct intracerebroventricular injection of excess FA into animal models causes memory decline [13–15]. Thus, it is necessary to determine the concentrations of endogenous FA in biological tissues.

To date, various analytical methods have been developed to detect endogenous FA. For instance, using a FA detection kit, a two-fold increase in the levels of FA (0.016 mM) was found in the cerebrospinal fluid of monkeys given an intracerebroventricular injection of methanol [31], which induced cognitive impairments, AD-like amyloid plaques, and tau hyperphosphorylation [30]. Using HPLC-Fluo, endogenous concentrations of FA in human blood and urine were found to be approximately 0.08 and 0.03 mM, respectively [12, 32]. Likewise, using HPLC-UV to determine levels of 2,4-dinitrophenylhydrazine, a derivative agent, showed that FA concentrations in the urine of normal Sprague-Dawley rats and humans were approximately 0.01 mM [33]. A method of headspace gas chromatography revealed that the concentrations of FA in human urine were between 0.019 and 0.048 mM [34]. Moreover, the endogenous FA concentrations in brain samples of control mice detected in the current study were approximately 0.27 mM, which is consistent with the previous reports using HPLC-Fluo [20], HPLC-UV [19], and GC–MS [17].

## 5. Conclusion

Using this modified Fluoral-P method, an abnormally high level of FA was detected in brain samples of FA-injected mice (Supplementary Figure 3). Taken together, the present spectrophotometric method using Fluoral-P as a derivative reagent for measurement of FA in brain samples constitutes a simple, sensitive, and practicable alternative to well-established methods for determining brain FA and assessing neurotoxicity of excess FA.

## Supplementary Material

Supplementary Fig. 1. Changes in the escape latency of FA-injected mice and control mice (A),the time in target quadrant (B), swimming distance in the target quadrant (C), and swimming traces in the target quadrant (D). Supplementary Fig.2. A yellow compound: 3,5-diacetyl-1, 4-dihydrolutidine (DDL) is immediately generated after the derivative reaction occurs between the colorless reagent- formaldehyde (FA) and 4-amino-3-pentene-2-one (Fluoral-P) at pH6.0. Supplementary Fig. 3. The spectrophotometric method is based on a raction in which formaldehyde (FA, HCHO) reacts with 4-amino-3-pentene-2-one (Fluoral-P) to produce a yellow compound:3,5-diacetyl-1,4-dihydrolutidine (DDL), and DDL can be detected by spectrophotometer at 420 nm. Using this method, an abnormal high level of FA is detected in the brain of FA-injected mice associated with spatial memory deficits (amnesia). Supplementary Table 1.

## Figures and Tables

**Table 1:** Concentrations of mice brain formaldehyde within a day at different time points.

Numbers	0 min	15 min	30 min	1 h	1.5 h	2 h	3 h
Sample 1	0.273	0.274	0.275	0.276	0.276	0.277	0.281
Sample 2	0.256	0.258	0.258	0.257	0.256	0.258	0.26
Sample 3	0.266	0.267	0.268	0.267	0.266	0.267	0.271
Sample 4	0.268	0.269	0.269	0.269	0.268	0.269	0.271
Sample 5	0.27	0.272	0.272	0.272	0.272	0.272	0.274
Sample 6	0.261	0.261	0.261	0.262	0.262	0.262	0.265
Sample 7	0.252	0.251	0.251	0.253	0.253	0.252	0.255
Sample 8	0.261	0.261	0.26	0.261	0.26	0.26	0.263
Sample 9	0.318	0.319	0.319	0.317	0.315	0.316	0.318
Mean ± S.D.	0.2694 ± 0.0064	0.2702 ± 0.0065	0.2703 ± 0.0065	0.2704 ± 0.0063	0.2698 ± 0.0061	0.2703 ± 0.0063	0.2731 ± 0.0061

Data in means (*n* = 9). No significant difference among the values within a day (*P* > 0.05).

**Table 2:** Changes in formaldehyde (FA) concentrations in the brains of C57 mice at different days.

Days	1 d	7 d	14 d	Mean	S.D.	RSD/%
Brain FA (mM)	0.2746	0.2787	0.2704	0.2746	0.0041	1.511

Data in means (*n* = 9). No significant difference among the values on different days (*P* > 0.05).

## References

[1] Salthammer T., Mentese S., Marutzky R. (2010). Formaldehyde in the indoor environment. *Chemical Reviews*.

[2] Tang X., Bai Y., Duong A., Smith M. T., Li L., Zhang L. (2009). Formaldehyde in China: production, consumption, exposure levels, and health effects. *Environment International*.

[3] Krzyzanowski M. (2008). WHO air quality guidelines for Europe. *Journal of Toxicology and Environmental Health, Part A*.

[4] Kilburn K. H., Warshaw R. H. (1992). Neurobehavioral effects of formaldehyde and solvents on histology technicians: repeated testing across time. *Environmental Research*.

[5] Kilburn K. H., Warshaw R. H., Thornton J. C. (1987). Formaldehyde impairs memory, equilibrium, and dexterity in histology technicians: effects which persist for days after exposure. *Archives of Environmental Health*.

[6] Usanmaz S. E., Akarsu E. S., Vural N. (2002). Neurotoxic effects of acute and subacute formaldehyde exposures in mice. *Environmental Toxicology and Pharmacology*.

[7] Liu Y., Ye Z., Luo H. (2009). Inhalative formaldehyde exposure enhances aggressive behavior and disturbs monoamines in frontal cortex synaptosome of male rats. *Neuroscience Letters*.

[8] Lu Z., Li C. M., Qiao Y., Yan Y., Yang X. (2008). Effect of inhaled formaldehyde on learning and memory of mice. *Indoor Air*.

[9] Taranenko N. A., Efimova N. V. (2007). Biomonitoring of formaldehyde in the urinary samples from the pediatric population in the Irkutsk Region. *Gigiena I Sanitariia*.

[10] Tong Z., Han C., Luo W. (2013). Accumulated hippocampal formaldehyde induces age-dependent memory decline. *Age (Dordrecht, Netherlands)*.

[11] Yu J., Su T., Zhou T. (2014). Uric formaldehyde levels are negatively correlated with cognitive abilities in healthy older adults. *Neuroscience Bulletin*.

[12] Tong Z. Q., Zhang J., Luo W. H. (2011). Urine formaldehyde level is inversely correlated to mini mental state examination scores in senile dementia. *Neurobiology of Aging*.

[13] Mei Y., Jiang C., Wan Y. (2015). Aging-associated formaldehyde-induced norepinephrine deficiency contributes to age-related memory decline. *Aging Cell*.

[14] Songur A., Ozen O. A., Sarsilmaz M. (2010). The toxic effects of formaldehyde on the nervous system. *Reviews of Environmental Contamination and Toxicology*.

[15] Gurel A., Coskun O., Armutcu F., Kanter M., Ozen O. A. (2005). Vitamin E against oxidative damage caused by formaldehyde in frontal cortex and hippocampus: biochemical and histological studies. *Journal of Chemical Neuroanatomy*.

[16] Tong Z., Han C., Qiang M. (2015). Age-related formaldehyde interferes with DNA methyltransferase function, causing memory loss in Alzheimer’s disease. *Neurobiology of Aging*.

[17] Heck H. D., White E., Casanova-Schmitz M. (1982). Determination of formaldehyde in biological tissues by gas chromatography/mass spectrometry. *Biomedical Mass Spectrometry*.

[18] Sardi E., Tyihak E. (1994). Simple determination of formaldehyde in dimedone adduct form in biological samples by high performance liquid chromatography. *Biomedical Chromatography*.

[19] Yilmaz B., Asci A., Kucukoglu K., Albayrak M. (2016). Simple high-performance liquid chromatography method for formaldehyde determination in human tissue through derivatization with 2, 4-dinitrophenylhydrazine. *Journal of Separation Science*.

[20] Tong Z., Han C., Luo W. (2013). Aging-associated excess formaldehyde leads to spatial memory deficits. *Scientific Reports*.

[21] Suzuki Y., Nakano N., Suzuki K. (2003). Portable sick house syndrome gas monitoring system based on novel colorimetric reagents for the highly selective and sensitive detection of formaldehyde. *Environmental Science & Technology*.

[22] Nash T. (1953). The colorimetric estimation of formaldehyde by means of the Hantzsch reaction. *The Biochemical Journal*.

[23] Meier C., Zünd F. (2000). Statistical methods in analytical chemistry. *Wiley-Interscience: New York*.

[24] Shrivastava A., Gupta B. (2011). Methods for the determination of limit of detection and limit of quantitation of the analytical methods. *Chronicles of Young Scientists*.

[25] Uhrovcik J. (2014). Strategy for determination of LOD and LOQ values—some basic aspects. *Talanta*.

[26] Teixeira L. S., Leao E. S., Dantas A. F., Pinheiro H. L., Costa A. C., de Andrade J. B. (2004). Determination of formaldehyde in Brazilian alcohol fuels by flow-injection solid phase spectrophotometry. *Talanta*.

[27] Khanmohammadi M., Dalali N., Karami F., Garmarudi A. B., Nemati H. (2012). Quantitative determination of formaldehyde by spectrophotometry utilizing multivariate curve resolution. *Bulletin of the Chemical Society of Ethiopia*.

[28] Rizak J. D., Ma Y., Hu X. (2014). Is formaldehyde the missing link in AD pathology? The differential aggregation of amyloid-Beta with APOE isoforms in vitro. *Current Alzheimer Research*.

[29] Yang M., Lu J., Miao J. (2014). Alzheimer’s disease and methanol toxicity (part 1): chronic methanol feeding led to memory impairments and tau hyperphosphorylation in mice. *Journal of Alzheimer's Disease*.

[30] Yang M., Miao J., Rizak J. (2014). Alzheimer’s disease and methanol toxicity (part 2): lessons from four rhesus macaques (Macaca Mulatta) chronically fed methanol. *Journal of Alzheimer's Disease*.

[31] Zhai R., Zheng N., Rizak J., Hu X. (2016). Evidence for conversion of methanol to formaldehyde in nonhuman primate brain. *Analytical Cellular Pathology (Amsterdam)*.

[32] Luo W., Li H., Zhang Y., Ang C. Y. (2001). Determination of formaldehyde in blood plasma by high-performance liquid chromatography with fluorescence detection. *Journal of Chromatography. B, Biomedical Sciences and Applications*.

[33] Deng Y., Yu P. H. (1999). Simultaneous determination of formaldehyde and methylglyoxal in urine: involvement of semicarbazide-sensitive amine oxidase-mediated deamination in diabetic complications. *Journal of Chromatographic Science*.

[34] Takeuchi A., Takigawa T., Abe M. (2007). Determination of formaldehyde in urine by headspace gas chromatography. *Bulletin of Environmental Contamination and Toxicology*.

