# Supplementary material for: A Sensitive and Rapid Method for Detecting Formaldehyde in Brain Tissues
Source: Anal Cell Pathol (Amst). 2017 Sep 24;2017:9043134. doi: 10.1155/2017/9043134 (PMC5632857; doi:10.1155/2017/9043134)
Supplement: Supplementary file 1 — Supplementary Fig. 1. Changes in the escape latency of FA-injected mice and control mice (A),the time in target quadrant (B), swimming distance in the target quadrant (C), and swimming traces in the target quadrant (D). Supplementary Fig.2. A yellow compound: 3,5-diacetyl-1, 4-dihydrolutidine (DDL) is immediately generated after the derivative reaction occurs between the colorless reagent- formaldehyde (FA) and 4-amino-3-pentene-2-one (Fluoral-P) at pH6.0. Supplementary Fig. 3. The spectrophotometric method is based on a raction in which formaldehyde (FA, HCHO) reacts with 4-amino-3-pentene-2-one (Fluoral-P) to produce a yellow compound:3,5-diacetyl-1,4-dihydrolutidine (DDL), and DDL can be detected by spectrophotometer at 420 nm. Using this method, an abnormal high level of FA is detected in the brain of FA-injected mice associated with spatial memory deficits (amnesia). Supplementary Table 1. [file 9043134.f1.pdf]

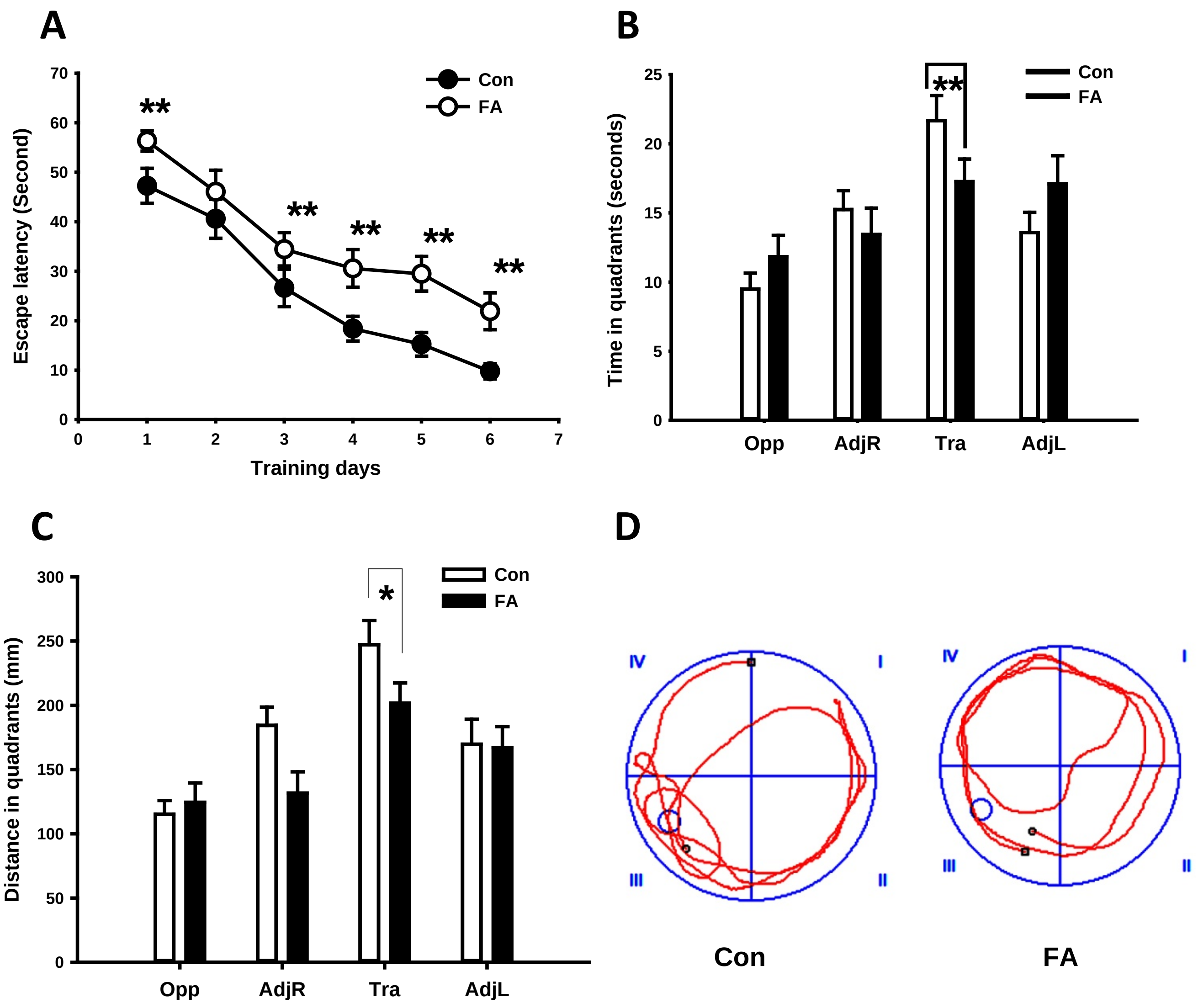

Supplementary Fig. 1. Changes in the escape latency of FA-injected mice and control mice (**A**), the time in target quadrant (**B**), swimming distance in the target quadrant (**C**), and swimming traces in the target quadrant (**D**).

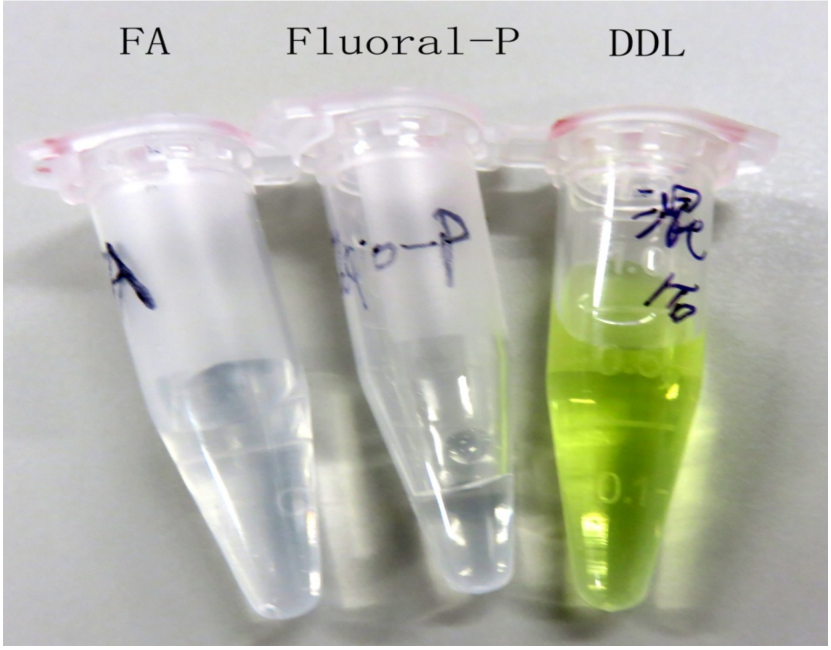

Supplementary Fig. 2. A yellow compound: 3,5-diacetyl-1,4-dihydrolutidine (DDL) is immediately generated after the derivative reaction occurs between the colorless reagent- formaldehyde (FA) and 4-amino-3-pentene-2-one (Fluoral-P) at pH 6.0.

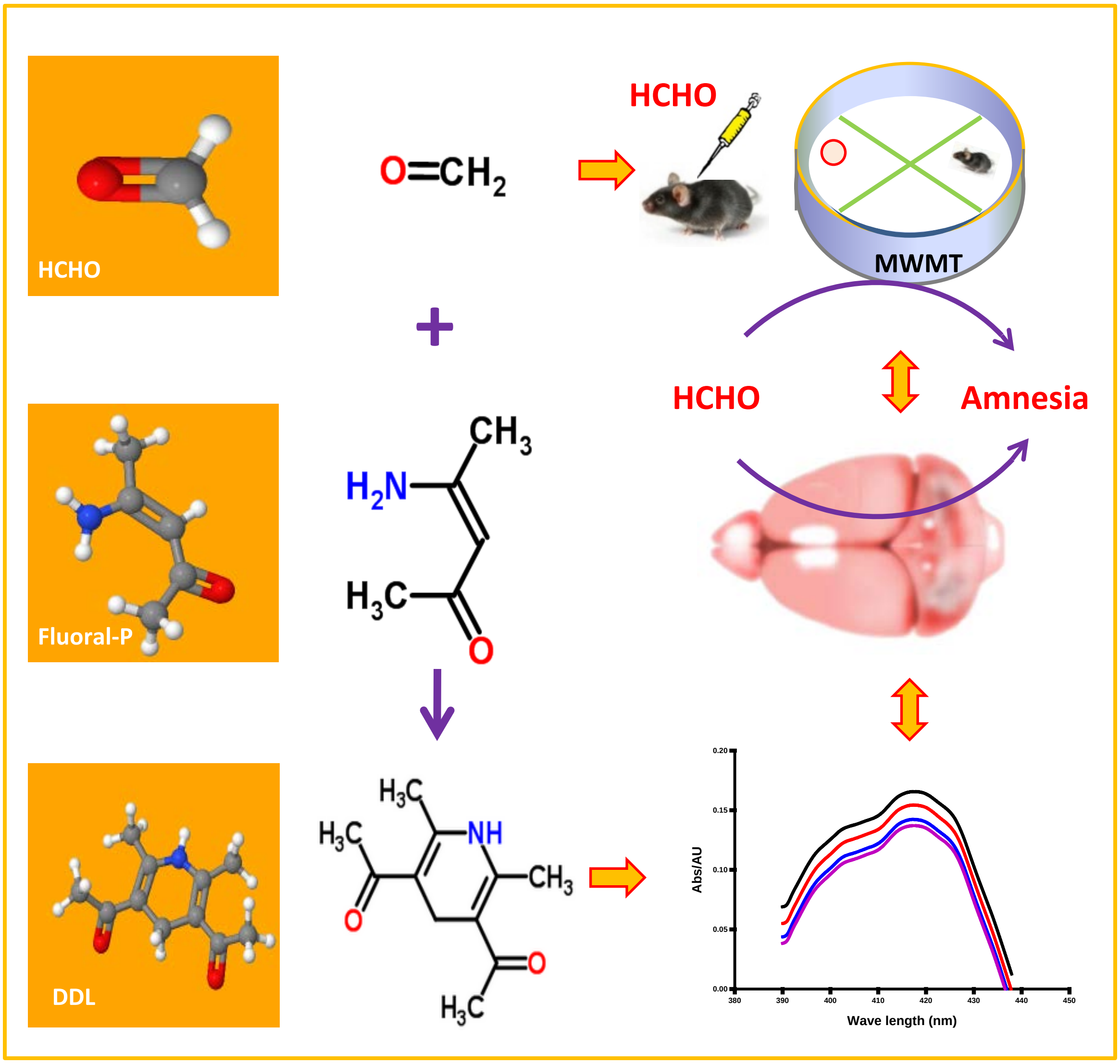

Supplementary Fig. 3. The spectrophotometric method is based on a reaction in which formaldehyde (FA, HCHO) reacts with 4-amino-3-pentene-2-one (Fluoral-P) to produce a yellow compound: 3,5-diacetyl-1,4-dihydrolutidine (DDL), and DDL can be detected by spectrophotometer at 420 nm. Using this method, an abnormal high level of FA is detected in the brain of FA-injected mice associated with spatial memory deficits (amnesia).

Supplementary Table 1

Table S1: Characteristics of autopsy samples from postmortem human hippocampus

	Alzheimer's disease Patients (n=9)	Age-matched controls (n=8)
Age (Years)	85.14±5.62	83.60±7.13
ApoE genotypes	ε 4/ ε 4	ε 3/ ε 3
Postmortem interval (h)	8.11±5.62	8.25±4.85
Brain pH	6.56±0.06	6.52±0.07
Amyloid	+++	----

** The age (years), postmortem interval (hours), and brain pH from autopsy hippocampal samples did not differ significantly between the respective groups. h: hour.
